# Female Genital Schistosomiasis: A Neglected among the Neglected Tropical Diseases

**DOI:** 10.3390/microorganisms12030458

**Published:** 2024-02-24

**Authors:** Benedetta Rossi, Letizia Previtali, Martina Salvi, Roberta Gerami, Lina Rachele Tomasoni, Eugenia Quiros-Roldan

**Affiliations:** 1 Unit of Infectious and Tropical Diseases, Department of Clinical and Experimental Sciences, University of Brescia and ASST Spedali Civili di Brescia, 25123 Brescia, Italy; l.previtali@studenti.unibs.it (L.P.); m.salvi026@unibs.it (M.S.); r.gerami@unibs.it (R.G.); maria.quirosroldan@unibs.it (E.Q.-R.); 2 School of Advanced Studies, Department of Experimental Medicine and Public Health, University of Camerino, 62032 Camerino, Italy; 3 Unit of Infectious and Tropical Diseases, ASST Spedali Civili di Brescia, 25123 Brescia, Italy; linatomasoni@yahoo.it

**Keywords:** female genital schistosomiasis, neglected tropical diseases, HIV, *Schistosoma haematobium*, schistosomiasis in pregnancy, schisto-IRIS, immunity, screening

## Abstract

Schistosomiasis is a neglected parasitic disease linked to water, posing a global public health concern with a significant burden in sub-Saharan Africa. It is transmitted by *Schistosoma* spp., causing both acute and chronic effects affecting the urogenital or the hepato-intestinal system. Through granuloma formation, chronic schistosomiasis weakens host immunity, heightening susceptibility to coinfections. Notably, female genital schistosomiasis (FGS), a disregarded gynecological condition, adversely affects girls’ and women’s reproductive health and increases vulnerability to HIV. This review explores the intricate interplay between schistosomiasis and HIV, considering their geographical overlap. We delve into the clinical features of this coinfection, underlying mutual influences on transmission, diagnostic challenges, and therapeutic approaches. Understanding the dynamics of FGS and HIV coinfection is pivotal for integrated healthcare strategies in regions with co-endemicity, aiming to mitigate the impact of the two infections on vulnerable populations.

## 1. Introduction

Female genital schistosomiasis (FGS) is a parasitic gynecological condition that is often underreported, misdiagnosed, and consequently largely untreated. Even though it affects up to 56 million women and young girls worldwide [1], only in 1997 the gender task force of World Health Organization’s Tropical Diseases Program included FGS into the scientific priority areas [2]. FGS represents a chronic manifestation of urogenital schistosomiasis, a water- and poverty-related-disease that exposes women to the risk of poor sexual and reproductive health, including complications during pregnancy or infertility, along with an increased susceptibility to acquire sexually transmitted diseases such as HIV [3].

Schistosomiasis is a neglected disease caused by the blood trematode *Schistosoma* spp. [4]. According to the World Health Organization (WHO), this parasitic disease affects almost 240 million people worldwide, with at least 90% living in sub-Saharan Africa (SSA). It is estimated that over 700 million individuals are at risk at any given time because they live in endemic tropical and sub-tropical areas in impoverished communities with limited access to clean water and adequate sanitation [5]. The highest prevalence of infection is observed among individuals aged 5–15 years: school-aged children, together with young girls and adolescents, are more susceptible to schistosomiasis due to their involvement in water-related activities [6,7].

Six major species in the genus *Schistosoma* significantly contribute to disease morbidity in humans: *S. mansoni*, *S. japonicum*, *S. intercalatum*, *S. guineensis*, and *S. mekongi* (mainly responsible for the hepato-intestinal form), and *S. haematobium* (for the urogenital form). Despite their varied geographical distribution, all species are contracted through direct contact with schistosome-contaminated freshwater inhabited by specific snails, the intermediate hosts [8,9,10]. Infection occurs during water-related activities such as personal hygiene, laundry, recreational practices, and agricultural work, alongside inadequate waste management systems [6].

*S. haematobium* accounts for two-thirds of global schistosomiasis cases, primarily distributed in SSA and the Middle East, where the pathogen is more prevalent [11]. Among others, *S. haematobium* is the primary species implicated in FGS (affecting 50–80% of women parasitized by this species); *S. mansoni* can also contribute to FGS [2]. Limited information is available regarding FGS caused by other species of schistosomes, necessitating further research [12].

*S. haematobium* adult worms live as pairs in pelvic venous plexuses within the infected human host: after mating, the female can produce hundreds of eggs per day [13]. Approximately half of these eggs are excreted in urine to sustain the parasite’s life cycle, whereas the remaining eggs become entrapped in capillary beds, inducing inflammatory reactions responsible for morbidity [4]. *S. mansoni*, *S. intercalatum*, *S. guineensis*, and *S. mekongi* occupy mesenteric venous plexuses of the bowel and rectum, and eggs are excreted in feces, while *S. japonicum* infection can be based both in the mesenteric and pelvic circulation [4].

Given that the silent epidemic of FGS affects the same population that bears a disproportionate global burden of HIV, this narrative review aims to highlight the characteristics of this coinfection, exploring mutual influences on transmission, clinical, immunological, and treatment features.

## 2. Pathogenesis of Schistosomiasis

Most schistosome infections often manifest as asymptomatic or paucisymptomatic forms (60%), owing to a successful host-parasite adaptation strategy. However, in around 10% of cases, infected individuals may develop symptoms due to chronic interactions between the pathogen and the host’s immune system [4].

Immunopathology predominantly arises from granuloma inflammation around eggs trapped in various organs [4]. Understanding human immune responses to this parasitic infection has largely been facilitated by experimental murine models of schistosome infection. *S. mansoni* has been the most extensively studied because its cycle in the murine model shares several characteristics with human infection. Conversely, experimental *S. haematobium* infections in mice do not involve the migration of adult worms to venous plexuses or the deposition of eggs in the bladder [14].

Recent reviews highlight that during the early phase of infection, the migration of schistosomula elicits a type 1 immune response characterized by heightened release of Interleukin (IL)-12 and interferon (INF)-g, persisting for approximately five weeks. As the parasite matures into the adult form and begins egg production, a shift toward a type 2 immune response occurs, marked by decreased INF-g production and a CD4^+^ T helper (Th) 2 polarization [15]. This shift in immunity leads to granuloma formation, resulting in diverse tissue reactions surrounding *S. haematobium* ova, ranging from marked periovular granulation tissue to irreversible fibrosis [16]. Schistosome infection can significantly influence cytokine levels. In the local Th2 environment, there is an expansion of CD4^+^ T-cells, along with increased production of cytokines like IL-4, IL-5, IL-13, which directly contribute to fibrogenesis and the polarization of macrophages toward the M2 phenotype [17,18]. Conversely, Dupnik et al. demonstrated a reduction in the expression of certain cytokines such as IL-15 due to schistosome infection [19].

Additionally, eggs induce neoangiogenesis around the site of ova deposition. Jourdan et al., comparing cervical histological samples from women with and without genital schistosomiasis, found significantly higher vascularization in the mucosa of women diagnosed with genital schistosomiasis compared to healthy cervical tissue from non-endemic controls [20]. Studies conducted on *S. mansoni* have revealed that both viable ova and soluble egg antigens (SEA) released by eggs stimulate the proliferation and migration of endothelial cells, inducing neovascularization [21]. In particular, Loeffler et al. investigated the role of SEA in angiogenic processes, demonstrating its capacity to induce proliferation, promote tube formation, and endothelial cell apoptosis. These effects seem to involve, at least in part, the upregulation of vascular endothelial growth factor (VEGF) expression in endothelial cells [22].

This immunological shift due to chronic schistosomiasis infections has been associated with a heightened risk of acquiring infections such as HIV or reactivation of latent infections like tuberculosis [23,24,25]. Additionally, undetected chronic parasitic infections might affect vaccine kinetics, inducing a faster decline in antibody levels over time or a lack of specific antibody production. Both primate models and human studies have observed inadequate responses to the HPV vaccine, Hepatitis B, and tetanus toxoid, respectively [26,27]. The coexistence of these diseases poses a significant threat in low-income countries where geographical overlap exists.

Furthermore, epidemiological evidence linking cancer and schistosomiasis was first put forward in 1984, suggesting that infection with *S. japonicum* might be a contributing factor to the development of colorectal cancer [28]. Subsequently, *S. japonicum* has also been linked to a higher risk of liver cancer, while *S. hematobium* has been linked to urinary bladder cancer. Presently, the International Agency for Research on Cancer (IARC) has associated schistosomal infection with colon cancer, liver cancer, and urinary bladder cancer [29,30], classifying schistosomes as a possible human carcinogen (*S. hematobium* group 1, *S. japonicum* group 2B, and *S. mansoni*, group 3, respectively) [31]. The pathogenesis involves complex inflammation-mediated mechanisms. However, evidence regarding the link between schistosomes and other cancers, such as prostate cancer, remains highly controversial. It is plausible that these associations could be coincidental [32].

Moreover, genital schistosomiasis might contribute to establishing persistent HPV infection, potentially contributing to the development of high-grade squamous intraepithelial neoplasia [33,34]. Cancers are an increasingly important cause of illness and death in people living with HIV (PLWH) especially those microorganism-related cancer [35,36]. No information is available about the risk of schistosome-related cancer in PLWH.

## 3. Clinical Manifestations

Schistosomiasis infection can manifest as both acute and chronic forms in humans. Acute manifestations encompass cercarial dermatitis, swimmers’ itch, and Katayama fever, all linked to systemic hypersensitivity reactions and immune complex formation against schistosome antigens. This acute phase often exhibits hypereosinophilia in peripheral blood, and serologic tests typically yield positive results in most cases [14,37].

In contrast, prolonged interaction between the host’s immune system and the parasite leads to chronic schistosomiasis, with different symptoms and clinical features based on the affected organs, in particular, hepato-intestinal, urinary, and genital organs.

In males, genital schistosomiasis results in granulomatous and fibrotic lesions within the local vascular system. Manifestations include prostatitis, orchitis, haemospermia, erectile dysfunction, hydrocele, phimosis, and oligo/azoospermia are the most frequent occurrences [38]. Contrary to this, the impact and consequences on the female genital tract are much more extensive.

### 3.1. Female Genital Schistosomiasis (FGS)

Female genital schistosomiasis is mainly caused by *S. haematobium*, although a few case reports reported have indicated the involvement of *S. mansoni* [39,40]. Adult worms migrate through the veins, draining pelvic organs and, via blood vessel anastomoses, reach genital organs, where females lay eggs. Among the gynecological sites affected, the cervix, fallopian tubes, and uterus are the most commonly observed (Table 1). Schistosomiasis can affect various organs, which is also due to parasite embolization [41]. Reported cases include ovarian localization due to female genital schistosomiasis caused by *S. mansoni* [41]. While the pathological mechanism of ovarian infestation remains unknown, it may be explained by the parasite’s ability to traverse extensively anastomosed abdominal and pelvic blood vessels [42]. It is also possible to have genital manifestations without urinary tract involvement, possibly due to individual variations in blood vessel patterns, blood flow, and worm localization [43].

Upon egg deposition in female genital tissue, chronic inflammation ensues, triggering symptoms and clinical features. The clinical pattern is characterized by nonspecific, painful, and stigmatizing symptoms such as leucorrhea, vaginal discharge, itching, contact bleeding, chronic abdominal pain, dyspareunia, and menstrual cycle abnormalities, all stemming from pathological changes in the genital mucosa [44,45]. Vulvar and perineal manifestations can encompass hypertrophic, ulcerative, fistulous, or wart-like lesions, often resembling other STIs. Internal lesions, detectable with colposcopy, typically present as sandy patches and rubbery papules [46]. Unrecognized and untreated female genital schistosomiasis can elevate the risk of acquiring STIs, including HIV. Additionally, it may lead to chronic inflammatory pelvic disease, subsequently causing salpingitis, infertility, ectopic pregnancy, and benign tumors such as cervical intraepithelial neoplasia (CIN) induced by *Schistosoma* [46,47].

**Table 1:** Gynecological sites affected by schistosome and associated signs and symptoms.

Localization of FGS	Percentage of Women with Gynecological Sites Affected by Schistosome	Publication Reference Number	Associated Signs and Symptoms	Publication Reference Number
Ovary	37.7% 33% 12.1%	[42] [48] [49]	Ovarian masses, lower abdominal pain, pseudo tumors	[41,50]
Fallopian tubes	24%	[42]	Infertility, sub-fertility, tubal adnexal masses, lower abdominal pain, ectopic pregnancy, salpingitis	[51,52,53,54]
	17%	[48]		
	4.3%	[49]		
Uterus	35.9%	[42]	Dysmenorrhea, menorrhagia, pelvic pain	[55,56]
	18%	[48]		
	15.5%	[49]		
Cervix	25%	[48]	Contact bleeding, dyspareunia, cervical polyps, cervical dysplasia, chronic cervicitis, infertility, sub-fertility	[24,49,57,58,59]
	54.3%	[49]		
Vagina	1%	[48]	Abnormal vaginal discharge, vaginal polyps, itching, polypous/papillomatous tumor, warts, recto-vaginal fistulas	[60,61,62,63]
Vulva	2.6%	[42]	Vulval itching, warts, plaques, bleeding pseudotumoural mass	[62,64,65]
	6%	[48]		
	5.2%	[49]		
Perineum	1.7%	[49]	Warts	[49]

In endemic areas, girls often acquire infection during childhood, with lesions manifesting later in life. Due to infrequent gynecological examinations, vulvar lesions are reported more commonly than vaginal and cervical lesions [46].

### 3.2. Schistosomiasis in Pregnancy

Roughly 40 million of women of childbearing age are estimated to be infected with schistosomes, with over 10 million women in Africa contracting the infection during pregnancy, mainly associated with *S. haematobium* [66]. Unfortunately, the literature lacks substantial data on infection prevalence among pregnant women and newborns [67,68,69].

The pathogenetic mechanisms of schistosomiasis in pregnant women remain unclear, but the infestation can cause ectopic pregnancy and miscarriage. *S. haematobium*, for instance, is linked to elevated levels of pro-inflammatory cytokines that cause cervix inflammation, potentially resulting in spontaneous miscarriage. Furthermore, inflammation of the fallopian tubes fosters the risk of ectopic pregnancy [49,70]. Studies suggest ectopic pregnancy may also be linked to impaired tubal motility due to mechanical obstruction from fibrotic granulomatous reactions or local ischemia due to egg deposition in the tube’s terminal veins [51,70]. These triggering factors can also determine infertility [52].

Moreover, common observations during schistosome infection include an increased risk of placental inflammation, interference with nutrient uptake, and maternal and fetal iron deficiency with consequent anemia [71]. Anemia results from pro-inflammatory cytokines production induced by schistosomes, which up-regulates hepcidin and decreases iron availability, compounded by iron loss due to eggs shedding through the bladder or gut walls [72]. Intestinal helminth infections during pregnancy, compromising nutritional status, may expose the fetus to reduced intrauterine growth (IUGR) [73]. To date, conflicting data exist regarding the association between IUGR, low birth weight delivery, and schistosome infection, necessitating further studies [74,75].

Furthermore, during pregnancy, progesterone and placental products cause an immunological shift to the Th2 immune response to avoid fetal rejection. This immunological change increases susceptibility to bacterial and viral infections, including exacerbating schistosome pathogenetic potential and its transmission [76,77].

Little is known about schistosome infection in pregnant women living with HIV, but more severe schistosomiasis symptoms are hypothesized, and further studies are needed [66].

## 4. Diagnosis

The diagnostic tools for schistosomiasis include direct methods such as detecting viable eggs on urine and feces specimens, detecting circulating antigens, employing schistosome genus-specific real-time PCR to detect parasite-specific DNA, and indirect methods like serological tests to identify specific antibodies [78]. Alongside laboratory tests, diagnostic procedures for schistosomiasis may include ultrasound, endoscopy, or biopsy [79].

FGS diagnosis is challenging, as there is not a widely accepted diagnostic reference standard for diagnosis and screening. Microscopy, the conventional diagnostic tool for schistosomiasis, has limitations for genital disease, as there is no direct correlation between the presence of *S. haematobium* eggs in urine and FGS. Its application on wet or pap smears has reported sensitivities of less than 15% [40,80]. So far, experts recommend the identification of specific mucosal findings using a colposcope or digital camera in people living in areas endemic to *S. haematobium*, which may suffice for FGS diagnosis. These findings include sandy patches appearing as single or clustered grains, homogeneous yellow areas, or rubbery papules [3,46,81]. Biopsy revealing parasite eggs in genital tissue is considered a reference standard [82]. However, due to invasiveness, limited access to well-equipped facilities, and trained personnel in low-resource settings, this approach is not routinely adopted in endemic areas. Moreover, theoretical concerns regarding HIV acquisition post-biopsy have restrained the acceptance of cervical biopsy in research setting [46]. These challenges contribute to the underreporting and oversight of this form of the disease.

Studies comparing various diagnostic approaches for detecting female genital schistosomiasis have been conducted across several African countries, comprising symptoms assessment, gynecological examination, and DNA extraction from genital samples. For instance, in Nigeria, the prevalence of FGS in four endemic communities was found to be 27.4% based on self-reported symptoms (87 out of 317 women tested). Among the 20 women who underwent gynecological examination and colposcopy, 70.0% displayed visible genital tract lesions [83]. In Zambia, a recent study aimed to validate community-based diagnosis of FGS using genital self-swabs compared to cervicovaginal lavage (CVL) performed by a trained midwife. The prevalence of FGS detected by genital self-swabs was 5.7%, whereas it was 2.7% with CVL lavage [84]. Another recent study conducted by Ursini et al. assessed the FGS prevalence in North-Western Tanzania by using PCR on samples obtained through self-collected or healthcare worker-collected genital specimens. The prevalence of FGS was higher when considering PCR results obtained from self-sampled swabs compared to operator-collected genital samples (respectively 5.2% and 4.7%) [85]. DNA detection through PCR analysis for schistosomes may serve as a valuable tool in FGS diagnosis due to its reproducibility, high specificity, and the feasibility of self-collection of genital specimens for testing.

## 5. Treatment

Schistosomiasis treatment primarily relies on praziquantel (PZQ), but often, variable multidisciplinary management, including invasive procedures, is required to deal with morbidities and complications [79]. PZQ is a drug effective only against adult worms but not eggs or young worms. Its minimal side effects and low resistance make it suitable for use during pregnancy, lactation, and childhood. PZQ is pivotal in preventing disease complications and minimizing lesions [86]. WHO recommends a single course of PZQ as the gold standard treatment for urogenital and hepato-intestinal schistosomiasis in endemic areas.

Mass drug administration (MDA) using PZQ has been in practice since 2001 for preventive chemotherapy. Recently updated guidelines broadened the eligibility for preventive chemotherapy to individuals aged 2 years and older, advocating for more frequent treatments, including biannual MDA, and a lowered prevalence threshold for annual preventive chemotherapy [87]. These interventions are critical in preventing the chronic implications of schistosomiasis and reducing associated morbidities.

To mitigate FGS complications and their stigmatizing impact on women in sub-Saharan Africa, regular treatment with PZQ outside primary school-based deworming programs is crucial. Raising awareness through educational activities is equally essential [88]. Additionally, women and girls in this region also face a compounded vulnerability to HIV. Interconnected relationships among these diseases suggest that managing one condition may alleviate adverse effects in the others. Each of these illnesses has a specific and established preventive measure: antiretroviral therapy and pre-exposure prophylaxis for HIV and praziquantel treatment for female genital schistosomiasis.

Even if data on the therapeutic effect of praziquantel on gynecological lesions and symptoms have not yet been fully elucidated, treating women with FGS prevents further egg deposition, offering potential benefits such as improved fertility [47]. Moreover, in a study conducted in Zimbabwe, Kjetland et al. suggested that women treated for schistosomiasis before the age of 20 had a significantly lower prevalence of sandy patches and contact bleeding than untreated women [44].

Schistosomiasis, along with HIV, is one of the most widespread infections globally, and an increasing number of individuals require specific treatment. The coinfection of schistosomiasis and HIV thus necessitates simultaneous treatment, making it crucial to evaluate the interactions between these two types of treatments. In a recent study, Mutiti et al. demonstrated that concentrations of PZQ are reduced fourfold when taken concurrently with an antiretroviral regimen containing efavirenz, one of the most commonly used molecules in antiretroviral regimens in low-income countries [89]. This interaction could impact the effectiveness of PZQ treatment and mass drug administration programs for schistosomiasis in PLWH.

## 6. Schistosomiasis and HIV Interactions

The global burden of schistosomiasis overlaps geographically with HIV. HIV remains a major global health concern but also a gender health problem, particularly affecting adolescent girls and young women, with approximately 4000 new HIV infections globally occurring weekly. In 2022, women and girls of all ages accounted for 63% of all new HIV infections in SAA [90]. For instance, in countries like Ethiopia, Nigeria, South Africa, Zambia, and Zimbabwe, adult HIV prevalence ranges from 15% to 28%, while the prevalence of schistosomiasis commonly exceeds 50% in high-risk rural communities. The prevalence of both infections might be even higher in data-deficient areas such as Madagascar or Mozambique [91]. Considering epidemiological, immunological, and pathophysiological data, FGS has been suspected as a risk factor for the horizontal transmission of HIV for over 25 years. However, it was only in 2006 that Kjetland et al. reported an association between FGS and HIV coinfection based on data from Zimbabwe [92].

Since 2006, several epidemiological studies and systematic reviews have consistently demonstrated that female genital schistosomiasis (FGS) can increase the risk of horizontal transmission of HIV by up to three times [93,94,95]. Additionally, a multivariate analysis conducted on the prevalence of *S. haematobium* infection and HIV in SSA countries indicated that each *S. haematobium* infection per 100 individuals correlated with a 2.9% relative increase in HIV prevalence [96]. Affecting host immunological and physiological responses, schistosomiasis alters the relationship between the host and HIV infection; therefore, in the last decades, more attention has been directed toward understanding the coinfection dynamics between schistosomiasis and HIV.

### 6.1. The Presence of Susceptible Host Cells

**Figure 1:**
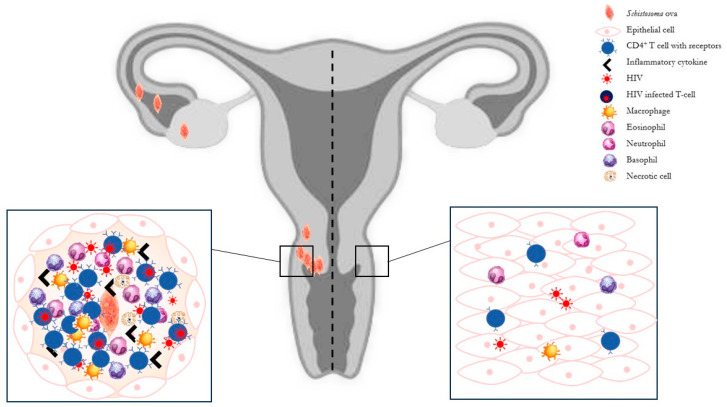
Representation of cellular populations in female genital organs without (to the **right** of the dashed line) and with schistosome infection (to the **left** of the dashed line). *S. haematobium* infection triggers a granulomatous response, with an increased CD4^+^ T-cells recruitment, cytokine and chemokine expression, and others’ inflammatory cell attraction. This condition increases the host cells’ susceptibility to HIV invasion.

During granuloma formation, CD4^+^ T-cells exhibit an increased expression of chemokine co-receptors and cellular CD4^+^ receptors, providing additional targets for HIV [97]. Specifically, the upregulation of the HIV coreceptors CC chemokine receptor 5 (CCR5) and CXC chemokine receptor 4 (CXCR4) on monocytes and lymphocytes facilitates the entry of HIV-1 into host cells [98]. Elevated concentrations of these receptors render cells more vulnerable to HIV invasion, thereby heightening the risk of HIV acquisition and viral spread in women with FGS [94,99] (Figure 1).

In a study conducted in South Africa, Kleppa et al. aimed to investigate the impact of schistosomiasis on HIV target cells within women’s genital tracts. Their findings highlighted a significant association between FGS and increased frequencies of CD14^+^ cells and higher frequencies of CD4^+^ T-cells expressing CCR5 in blood. Additionally, they observed a significant reduction in the CD4^+^ T-cell population expressing CCR5 in genital samples and in blood following anti-schistosomal treatment with PZQ [100].

### 6.2. Impaired Cervicovaginal Barrier

An intact vaginal barrier plays a pivotal role in influencing the vulnerability of hosts to HIV infection. Components such as an intact vaginal mucus layer, antimicrobial peptides, an acidic pH, an optimal vaginal microbiota, and a protected cervicovaginal epithelium collectively form the primary defense against HIV acquisition [101]. Much like other sexually transmitted infections (STIs), the distinctive lesions of FGS may serve as a potential entry point for HIV, breaching the cervicovaginal mucosal barrier [39,102].

The abnormal formation of new blood vessels around trapped ova weakens the vaginal mucosa, compromising its physical barrier role. This situation often leads to bleeding during sexual intercourse in women with FGS, facilitating the penetration of HIV into deeper genital cell layers through semen [94,103].

Furthermore, the pathological changes induced by FGS may exacerbate HIV shedding and heighten the transmission of HIV from HIV-positive women to HIV-negative men. Unlike many bacterial STIs that often heal after adequate treatment, several FGS-related genital sores persist even after the administration of PZQ [104,105].

### 6.3. The Role of Cervicovaginal Inflammation

*S. haematobium* infection has been shown to influence cytokine expression both systemically and in the vaginal environment [19,106,107]. Elevated chemotactic and inflammatory cytokine concentrations in the vagina can disrupt the expression of proteins associated with cervicovaginal epithelium integrity, potentially increasing susceptibility to HIV infection [108,109].

Conversely, decreased levels of certain cytokines, like IL-15, may increase susceptibility to infections, as IL-15 plays a crucial role in activating natural killer cells, as well as in the survival and proliferation of mononuclear cells and CD8^+^ T-cells [19].

### 6.4. The Role of HIV on Schistosome

While strong evidence exists indicating an increased risk of HIV acquisition in individuals infected with schistosomes, limited data are available regarding the role of HIV in schistosome infections [93]. Some evidence suggests that HIV might accelerate the progression to portal vein fibrosis in people infected with *S. mansoni* [110]. However, there is a lack of further studies elucidating the impact of HIV on urogenital lesions associated with schistosomiasis.

**Figure 2:**
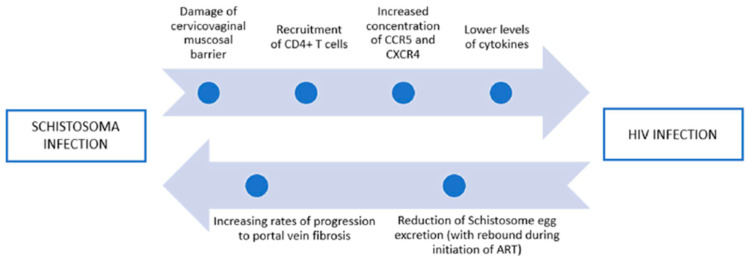
The role of schistosome infection on HIV and the role of HIV infection on schistosomiasis. CCR5, coreceptors CC chemokine receptor 5; CXCR4, chemokine receptor 4; IL, interleukin; ART, antiretroviral therapy.

On the other hand, consistent evidence indicates that HIV can reduce the excretion of schistosome eggs, which might rebound during the initiation of antiretroviral therapy [111,112] (Figure 2).

### 6.5. Schisto-IRIS

HIV-associated immune reconstitution inflammatory syndrome (IRIS) refers to the clinical worsening of an opportunistic infection, often involving tissue-destructive inflammation, after the start of antiretroviral therapy (ART). While the vast majority of pathogens associated with IRIS are mycobacterial, chronic viral, and invasive fungal infections, the co-endemic nature of schistosomiasis and HIV has raised the possibility of IRIS linked to schistosome infection [113]. There is no univocal consensus on schistosome-associated IRIS (schisto-IRIS) definition because this condition is still misdiagnosed and underreported.

Schisto-IRIS can be defined as either the re-emergence of symptoms of previously effectively otherwise successfully treated chronic schistosomiasis upon initiating ART or the production of schistosome eggs by individuals who were not producing them before starting ART [114,115]. The underlying immunological dysregulation in schisto-IRIS patients remains poorly understood. A 2014 study in Kenya by Ogola et al. found that among HIV patients previously treated for hepato-intestinal schistosomiasis, 36.6% developed schisto-IRIS. This study identified genetic variants within the IL-23R gene, both synonymous and non-synonymous, as potential predisposing factors for susceptibility to schisto-IRIS [114]. Another hypothesis suggests that schisto-IRIS may involve an excessive presence of pro-inflammatory factors post PZQ treatment, which typically boosts resistance to reinfection.

Drawing parallels from what is known about the acute inflammatory reaction to TB antigens in TB-IRIS, which involves cytokines storm, monocyte activation, and elevated levels of matrix metalloproteinases (MMPs) [116,117], Goovaerts et al. investigated the role of these markers in schisto-IRIS. MMPs and tissue inhibitors of metalloproteinases (TIMPs) play a role in inflammation, granuloma formation, and tissue remodeling. Human studies in the context of schistosomiasis associate MMP-1 and -2 and TIMP-1 and -2 with active periovular granulomas [118]. Goovaerts et al. evidenced that schisto-IRIS is characterized by an unbalanced MMP/TIMP dynamic that favors inflammation, persisting also after PZQ treatment [119].

Even though data remain limited, a high pre-ART schistosome antigen load and a short time interval between ART and treatment for the opportunistic infection could be recognized as potential risk factors for the occurrence of schisto-IRIS.

## 7. Conclusions and Future Directions

Schistosomiasis, a neglected disease, typically occurs in tropical and sub-tropical regions, primarily affecting the poorest communities, where it is more easily identified and potentially treatable. While cases are predominantly found in endemic areas, sporadic occurrences have been reported in Europe, specifically in Corsica, France, and Almeria, Spain [120,121]. The idea that climate change and globalization could contribute to the emergence of new instances of indigenous infection in non-endemic areas in the future has not been ruled out [122].

Currently, the heightened human migration from endemic to non-endemic regions increases the risk of misdiagnosing schistosomiasis, especially FGS, due to limited awareness in non-endemic countries. Additionally, existing schistosomiasis control strategies do not address FGS [123]. Recent research has revealed a high prevalence of schistosomiasis among African refugees and asylum seekers in Italy, but information on FGS in this population is lacking [124].

There is a need for enhanced education and information dissemination on FGS among healthcare professionals and urgent action is required to establish FGS control guidelines to bring female genital schistosomiasis out of neglect around the world [125]. Considering the link between FGS, HIV, and cervical cancer, adopting an integrated approach with comprehensive screening for these infections offers a chance to reach more girls and women throughout their lives. Furthermore, through a three-way interaction among these diseases, controlling one may reduce the risk of adverse outcomes for the other two.

## Data Availability

Not applicable.
